# OAGB with shortened excluded ileal loop as an effective treatment for type 2 diabetes mellitus in the cases of Caucasian men and women with obesity of the first degree (BMI 30–35 kg/m^2^)

**DOI:** 10.1007/s00423-023-02785-9

**Published:** 2023-02-11

**Authors:** Paweł Jaworski, Artur Binda, Krzysztof Barski, Karolina Wawiernia, Emilia Kudlicka, Michał Wąsowski, Piotr Jankowski, Wiesław Tarnowski

**Affiliations:** 1 Department of General, Oncological and Bariatric Surgery, Centre of Postgraduate Medical Education, Orłowski Hospital, Czerniakowska 231, 00-416 Warsaw, Poland; 2 Department of General Medicine and Gerontocardiology, Centre of Postgraduate Medical Education, Orłowski Hospital, Czerniakowska 231, 00-416 Warsaw, Poland

**Keywords:** Obesity, Bariatric surgery, Metabolic surgery, Type 2 diabetes, One anastomosis gastric bypass

## Abstract

**Introduction:**

The aim of the study is to assess the effect of shortening the excluded loop of the small intestine to 150 cm on the effectiveness of one anastomosis gastric bypass (OAGB) in remission of type 2 diabetes with I^o^ obesity.

**Material and methods:**

The study included 25 patients with a body mass index (BMI) 30–35 kg/m^2^, with a diagnosis of diabetes mellitus type 2 (T2DM), and undergoing OAGB with excluded 150 cm of the small intestine.

**Results:**

There were no deaths in the study group, bleeding during the postoperative period requiring reoperation, anastomotic leakage/leakage throught mechanical stitching. The mean a glycated haemoglobin (HbA1C) level 12 months after surgery was 6.16 ± 0.96%, corresponding to a 2.29 ± 3.3% decrease. In more than 85% of the patients taking insulin before surgery, the insulin was discontinued in the postoperative period. Additionally, the level of glycaemia was assessed in patients on the day of surgery (163 ± 58 mg/dl) and on the day of discharge from the hospital (4.7 ± 1.3 days)—it was lower by over 18% (133 ± 39.2 mg). Over the period of 12 months following OAGB, there was a reduction in the mean BMI value from 33.5 ± 2 to 25.5 ± 2.5 kg/m^2^ and improvement in lipid parameters and mean values of blood pressure.

**Conclusion:**

OAGB with excluded 150 cm of the small intestine has beneficial effect on the remission of T2DM in patients with a BMI of 30–35kg/m^2^ and is associated with an acceptable level of complications. The achieved weight loss after surgery is satisfactory.

## Introduction

In 2016, more than 1.9 billion adults aged 18 and older were overweight (representing 39% of the population), and of these individuals, more than 650 million were obese (13% of the population) [1]. Compared with 1975, the number of obese patients worldwide had tripled [2]. Overweight and obesity are major risk factors for many chronic diseases, including type 2 diabetes, hypertension, coronary heart disease, and cancer, as well death caused by cardiovascular disease. Once considered a problem of highly developed countries, obesity is now common worldwide [3].

Type 2 diabetes is a chronic disease caused by an inherited and/or acquired deficiency of insulin by the pancreas or ineffectiveness of the insulin produced. Such a deficiency causes an increase in blood glucose levels, which in turn damages many body systems, particularly blood vessels and nerves [4].

In patients with type 2 diabetes, both insulin action (insulin resistance) and insulin secretion are impaired, with one of these abnormalities playing a predominant role, depending on the stage of the disease [4].

According to a World Health Organization (WHO) report released in 2016, there are currently 422 million people with diabetes worldwide (90% have type 2 diabetes) [5]. The number of patients has already far exceeded the International Diabetes Federation's (IDF) 2009 projections of 380 million patients as late as 2025 [6].

On 20 December 2006, the United Nations General Assembly recognised diabetes as one of the 10 most important chronic diseases in the world by resolution 61/225 [7].

Current treatments for type 2 diabetes include diet, exercise and oral antidiabetic drugs; for some patients, insulin therapy is required over time [8]. The effectiveness of these methods compared with surgical treatment is limited. Surgical treatment combined with Intensive Medical Therapy (IMT) is more effective than intensive conservative treatment alone [9]. Schauer et al. classified patients with type 2 diabetes into one of three groups: Roux-en-Y gastric bypass (RYGB)+IMT, laparoscopic sleeve gastrectomy (LSG)+IMT and IMT. The end point of the study was a glycated haemoglobin (HbA1c) of 6%. One hundred fifty patients with type 2 diabetes were included in the study, follow-up lasted 5 years. During this time, one patient died and 90% (134 people) of the remaining patients enrolled in the study were evaluated. Of the 134 patients enrolled in the study, 49 underwent RYGB, 47 underwent LSG, and 38 patients received modified conservatively therapy (IMT). The mean HbA1c was 9.2 ± 1.5%, the mean BMI was 37 ± 3.5 kg/m^2^, and the majority (66%) of the patients included in the study were women. After 5 years, an HbA1C of less than 6% was reported in 29% (RYGB), 23% (LSG), and 5% (IMT), respectively. Comparing the group treated with surgery vs. the conservatively treated group had a 7-fold greater reduction in glycated hemoglobin (2.1% vs. 0.3%). Patients who had diabetes for less than 8 years and who were randomized to undergo restrictive-exclusion surgery were more likely to reach the study end point.

One of the latest methods of dealing with type 2 diabetes is metabolic surgery. It has been found that gastric bypass in patients with type 2 diabetes improves glucose tolerance. The exact mechanism of the operation contributing to the recovery from type 2 diabetes is not fully understood. There are several theories including the incretin theory, which suggests the mechanism of postprandial secretion of incretin hormones with simultaneous suppression of anticretin hormones [10].

Metabolic surgery is currently recommended for patients with BMI ≥ 40 kg/m^2^ regardless of glycaemic control and for patients with BMI ≥35 kg/m^2^ who are not well controlled on medications. In its guidelines, the American Diabetes Association (ADA) also emphasises that metabolic surgery should be considered in patients with a BMI of 30–35 kg/m^2^ and inadequate glycaemic control. The inclusion of metabolic surgery in the management of type 2 diabetes in widely accepted evidence-based guidelines will both clinically benefit patients [11]. The preferred methods for patients with type 2 diabetes are exclusion and restriction-exclusion surgery.

OAGB is effective in treating type 2 diabetes, but is associated with certain complications due to the length of the disabled bowel loop [12].

When planning this study, we wanted to find an answer to the question: Will shortening the bowel loop, which reduces the risk of deficiency complications, be equally effective in treating type 2 diabetes ?

## Materials and methods

In 2014–2018 twenty-five Caucasian men and women patients with a BMI of 30–35 kg/m2 and diagnosed type 2 diabetes (duration <10 years) were qualified for the study. They underwent surgery and were subjected to postoperative follow-up 12 months after the operation. No control group was formed due to the nature of the study and the parameters evaluated. To exclude latent autoimmune diabetes of adults (LADA) type diabetes an anti-glutamic acid decarboxylase (anti-GAD) antibody test was performed as well as a C-peptide level determination on fasting (>1 ng/ml) and after glucagon loading to define pancreatic reserves of insulin.

Polish recommendations in bariatric and metabolic surgery have been developed based on the guidelines American Diabetes Federation (ADA), European Guidelines for Obesity Management in Adults, American Association of Clinical Endocrinologist, The Obesity Society, American Society for Metabolic and Bariatric Surgery (AACE, TOS, ASMBS), International Diabetes Federation (IDF) and European Association for Endoscopic Surgery (EAES) guidelines. [13]. Qualification for surgical bariatric/metabolic treatment is one of the key factors influencing its outcomes. Before offering the patient a surgical treatment, we analyze the previous history of attempts to reduce weight and prequalify the patient for surgery. The final qualification of the patient for surgery is multidisciplinary: it is a decision of a team consisting of specialists experienced in obesity treatment, namely, a surgeon, internist, anaesthesiologist, psychologist or psychiatrist , dietician, physiotherapist, and, if necessary, a cardiologist, pulmonologist, gastroenterologist and neurologist. Recommendations also define the optimal time of patient preparation for surgery, it should not be shorter than 3 months or longer than 6–12 months.

### Inclusion criteria for participation in the study

1. Age 18–65 years

2. BMI 30–35 kg/m^2^

3. Type 2 diabetes mellitus lasting less than 10 years

4. Negative test for anti-GAD antibodies

5. Serum C-peptide level greater than 1 ng/ml, increase in C-peptide concentration after stimulation with glucagon (glucagon test)

6. Informed written consent was obtained for surgery and participation in the study.

### Exclusion criteria for participation in the study

1. Age > 65 or <18 years

2. General contraindications for general anaesthesia or major surgery

3. Diseases requiring psychiatric treatment

4. Neoplastic disease

5. Inflammatory bowel diseases

6. Addiction to alcohol, drugs or medications

7. Secondary obesity

6. Lack of informed/written consent for surgery or participation in the study

7. Serum C-peptide concentration <1 ng/ml or no increase after glucagon loading

8. Positive test for anti-GAD antibodies

### Stages of operation

**Fig. 1:**
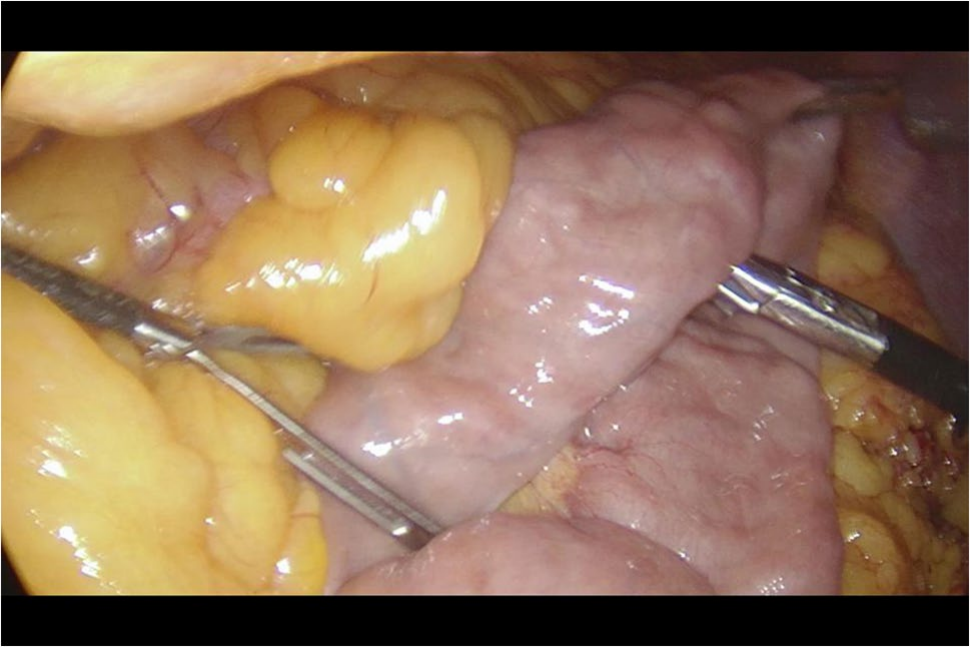
Measuring the jejunum loop

An extremely important element of the described operation is the length of the intestinal loop excluded from passage, due to the malnutrition syndromes described in the world literature after this operation, we decided to exclude 150 cm of the small intestine (Fig. 1), which is one of the innovative elements of this work.

### Surgical technique

**Fig. 2:**
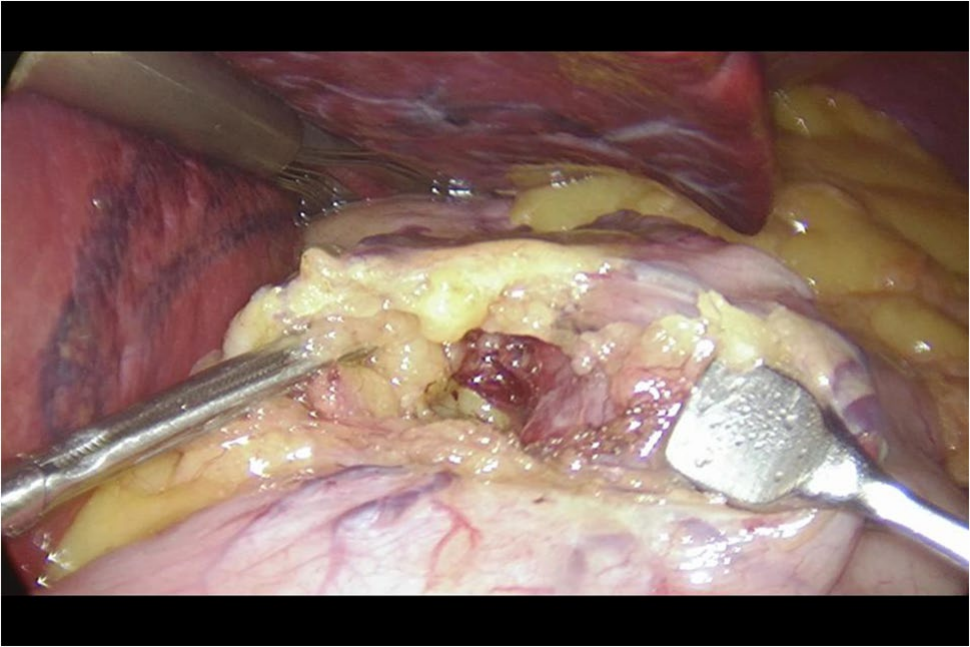
Cutting a lesser omentum

**Fig. 3:**
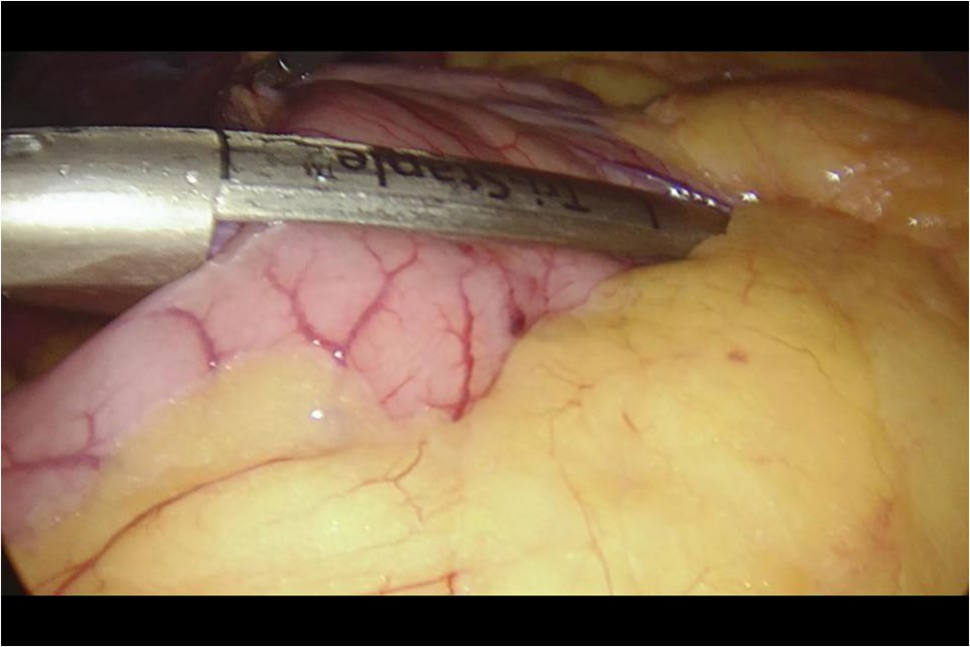
Transverse gastric dissection

**Fig. 4:**
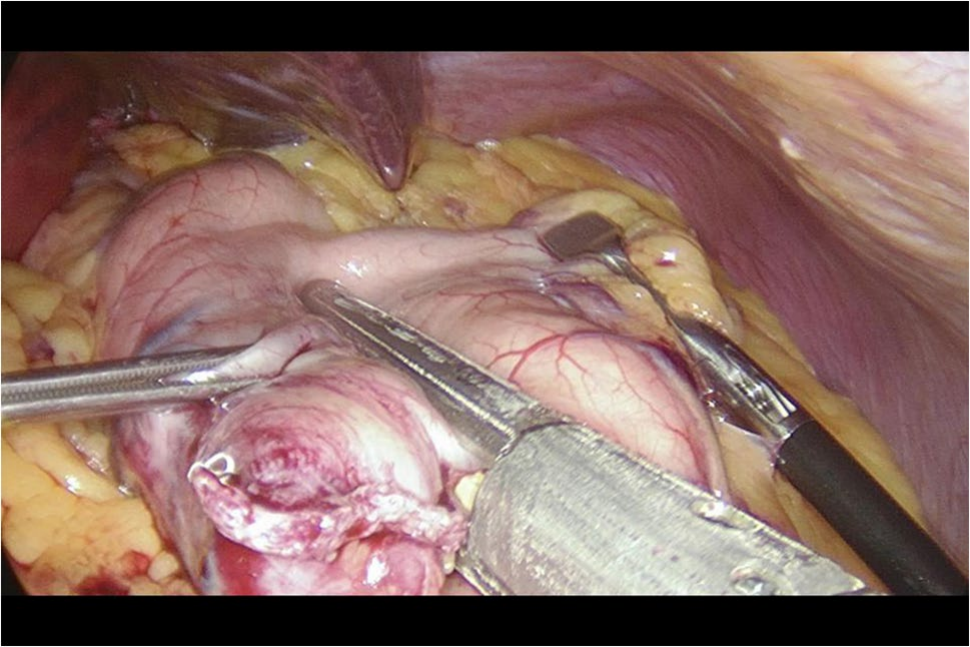
Vertical intersection of the stomach

**Fig. 5:**
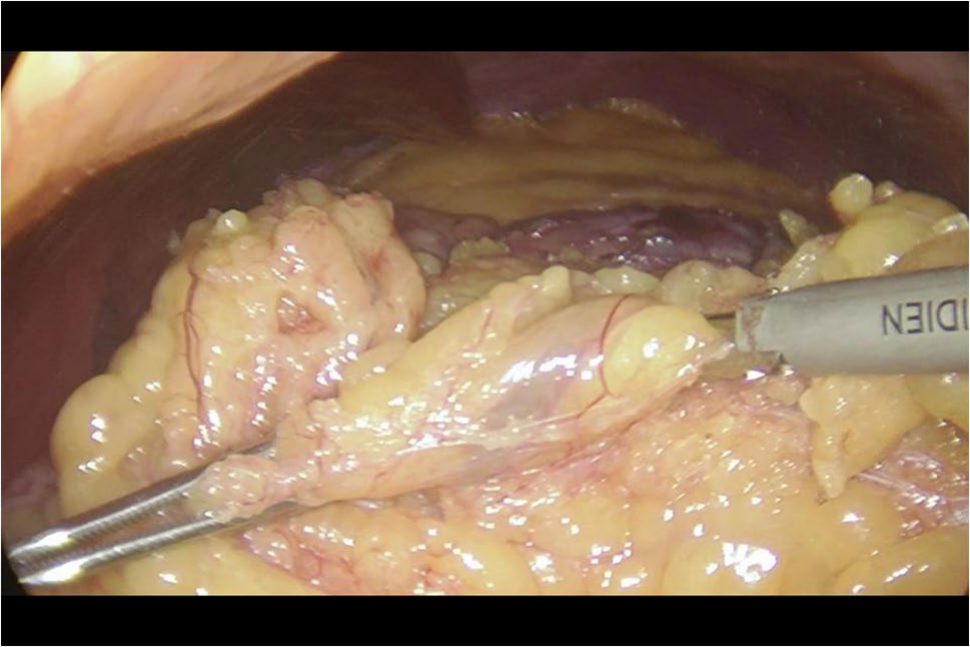
Cutting a greater omentum

**Fig. 6:**
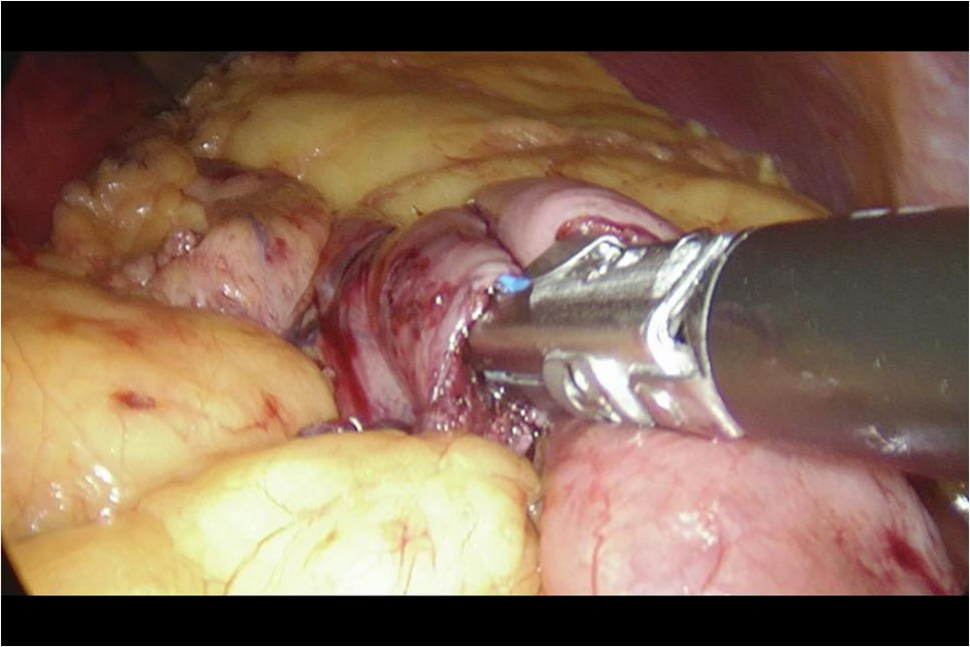
Performing a gastrointestinal anastomosis

**Fig. 7:**
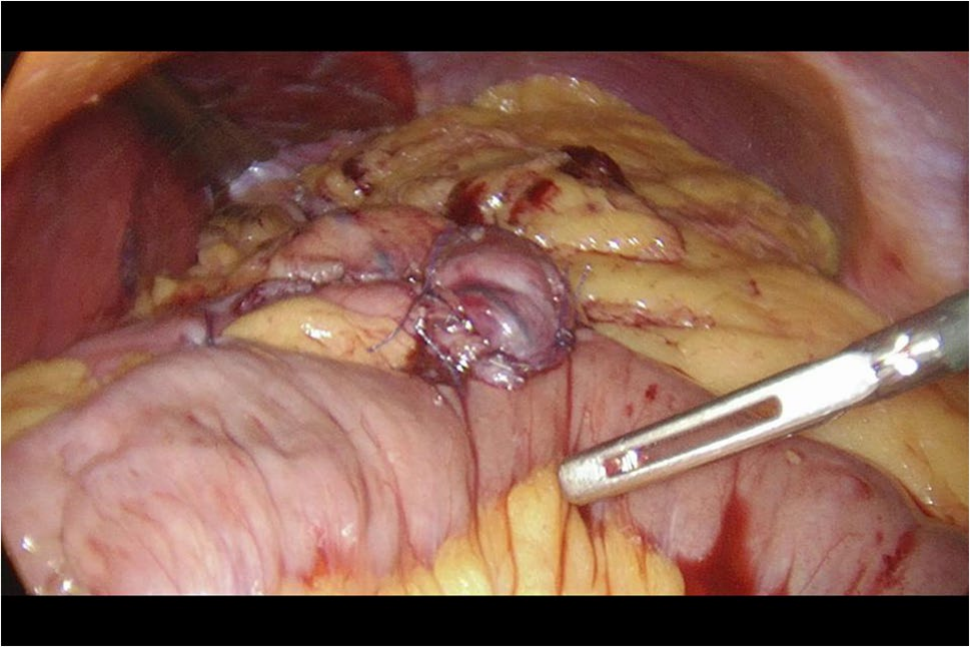
OAGB—view after surgery

Written informed consent was obtained from the patients a day before the surgery. Prior to the operation, each patient received anticoagulation and antibiotic prophylaxis according to the current bariatric guidelines. After inducing general anaesthesia, the patient was placed in a French setup in an anti-Trendelenburg position. The operation was initiated by generating a pneumothorax with the use of a Veress needle, puncturing it at the Palmer's point under the left costal arch. The first trocar, optical, was inserted halfway between the xiphoid process and the navel, slightly to the left of the midline. The next two 12–15 mm trocars were inserted approx. 5 cm to the left and 5 cm to the right of the optical trocar, generally at the same level. Through the fourth trocar, which was placed just below the xiphoid process and slightly to the right of the midline, a retractor was inserted, which raised the left lobe of the liver. The fifth trocar was placed under the left costal arch in the anterior axillary line. After carefully examining the peritoneum and identifying the Treitz ligament, 150 cm of the jejunum was measured (Fig. 1) (we used a laparoscopic instrument with a marker placed on it to measure the intestine). Then, the area of the gastroesophageal junction and Hiss angle were located, and in the next stage the gastro-diaphragmatic ligament was cut. This manoeuvre significantly facilitates the preparation of the posterior gastric wall in subsequent stages of the operation and allows for the safe creation of a window behind the posterior gastric wall. Next, we identified the minor curvature of the stomach and the crow’s foot (junction of body and antrum of the stomach). At this height, as close as possible to the stomach wall, the preparation of the gastro-hepatic ligament began and the lesser sac was opened (Fig. 2) to gain access to the back wall of the stomach. Then, the Endo-GIA 45 mm stapler was inserted through the trocar located in the right mesogastrium and crossed the stomach (Fig. 3). After the transverse section of the stomach, 36 French the nasogastric tube was inserted into the stomach and the stomach was cut vertically towards the Hiss angle. Approximately 4–5 staples were used to create a neo-stomach with a volume of approximately 20 ml (Fig. 4). The next stage of the operation is the vertical cutting of the greater omentum (Fig. 5). For the anastomosis, a 45-mm blue stapler was used (Fig. 6). The anastomosis should not be wider than 2–3 cm. The stapler defect was closed with an absorbable suture. An important element that needs to be considered at this stage is the correct positioning of the small intestine loops. An anastomosis of the intestine should be made to the end of the gastric stump, and the intestinal loop should be sutured to the excluded part of the stomach in such a way that the direction of the loop runs downward to reduce the possibility of reflux of the intestinal contents into the gastric pouch. The distal segment of the intestinal loop was sewn to the antral area to reduce the tension in the anastomosisand to serve as part of the antireflux mechanism Anastomosis tightness was assessed with the methylene blue test. A drain was placed next to the anastomosis (Fig. 7).

### Postoperative care

On the first postoperative day, the patients underwent the methylene blueoral test, and no leakage was observed when a liquid diet was administered. Good oral diet tolerance and satisfactory well-being of the patient signalled the possibility of discharge. All of the patients received diet recommendations, vitamin and mineral supplementation and follow-up schedule.

After the operation, constant supervision is necessary (by the coordinator of bariatric patients), along with monitoring of obligatory control visits, analysis of examination results, and participation of patients in support group meetings (both before and after surgery).

### Scheme of follow-up visits during the observation period

- the day of hospital discharge after surgery
- 4–6 months after surgery
- After 12 months there is a reassessment of the general condition, evaluation of the possible presence of complications, evaluation of weight reduction parameters, concomitant diseases, and changes in pharmacotherapy of type 2 diabetes and other concomitant diseases, along with the assessment of HbA1c (glycated haemoglobin) during hospitalization for 3–4 days.

The protocol presented above is related to planned visits, and the patient has constant contact with the centre performing the surgery.

The values used to assess the remission or improvement of type 2 diabetes and concomitant diseases of obesity assessed in the study 12 months after surgery were adopted according to the criteria of the Polish Diabetes Association (Clinical recommendations for the management of patients with type 2 diabetes):

- Remission: HbA1c value is <6.5% without pharmacotherapy, weight loss is > 15% compared to with the state at the time of qualification for surgery
- Improvement: HbA1c is reduced by > 20% after reducing the number of doses of medications taken before surgery

### Statistical analysis

Statistical analyses were conducted using the IBM SPSS Statistics package version 24, with which basic descriptive statistics were calculated, a series of Mann-Whitney *U*, Wilcoxon tests, a series of correlation analyses using Spearman’s rho coefficient and linear regression analysis were performed. The level of significance in this study was taken to be *α* = 0.05, while results of 0.05 < *p* < 0.1 were considered significant at the level of statistical trend.

## Results

The analysis included the obtained results of 13 women and 12 men during the observation period. The average age of the patients (*n*=25) was 50.6±9.5 years. The average HbA1c level before surgery was 8.45±3.3 % and 12 months after surgery it was 6.16±0.96%, corresponding to a decrease of 2.29±3.3%.

According to the adopted criteria, remission of type 2 diabetes was observed among 14 patients (56%) and improvement was observed in 6 patients. From the group of 25 patients: 21 patients were taking insulin and/or oral hypoglycaemic medication, 3 patients were only on oral medication therapy and 1 patient was taking Glucagon-like peptide-1 (GLP1) analogues. More than 85% of patients taking insulin before surgery discontinued it in the postoperative period. In addition, the patients’ glycaemic levels on the day of surgery (163 ±58 mg/dl) and on the day of discharge from the hospital (4.7±1.3 days) were assessed, and they were more than 18% (133 ±39.2 mg/dl) decreased at discharge. The average glycaemia 12 months after surgery was 107±17 mg/dl (Table 1).

**Table 1:** Glycemic values on the day of surgery, hospital discharge, and follow-up

	On the day of the operation	On the day of discharge from hospital (postoperative day)	12 months after operation
	Average value	Average value	Average value
Glycemia (mg/dl)	163±58	133±39 (4.7±1.3)	107±17

In addition to assessing the recovery from type 2 diabetes, the reduction in body weight and associated diseases was also assessed. The average body weight of patients undergoing OAGB was 97.1±11.6 kg and 73.8±12.2 kg at 12 months after surgery, representing a reduction in the mean BMI from 33.5±2 kg/m^2^ to 25.5±2.5 kg/m^2^ (Table 2).

**Table 2:** Comparison of weight reduction parameters

	Before operation (*n*=25)	Assessment of 12 months after operation (*n*=25)
	Average value	Average value
BMI (kg/m^2^)	33.5±2	25.5±2.5
Body weight (kg)	97.1±11.6	73.8±12.2
% excess weight loss (EWL)	102.7±38.5	
% total weight loss (TWL)	24.6±5.6	

The average blood pressure values at the time of study qualification and the 12-month follow-up were 131±15.5/78±7.6 mm Hg and 116±8/71±8.3 mm Hg, respectively. In the group of 25 patients, there were 16 people with diagnosed arterial hypertension, 8 of them in the postoperative period had discontinued antihypertensive drugs with complete normalization of blood pressure (complete remission), and 5 patients improved in terms of treatment (reduction in intake of hypertensive drugs).

A follow-up visit (hospitalization) 12 months after the operation also confirmed improvement in lipid parameters, as follow: preoperative total cholesterol 178.8±42.2 mg/dl, HDL 37. 9±12.5 mg/dl, LDL 102.8±39.6 mg/dl, triglycerides 193.8±66.1 mg/dl, postoperative total cholesterol 167.2±36.2 mg/dl, HDL 49.73±13.76 mg/dl, LDL 92±30.9 mg/dl, triglycerides 127.2±72.55 mg/dl. This trend had already been observed during previous follow-up visits to the Surgical Clinic. Among the 14 patients with lipid disorders, 6 stopped taking medication in the postoperative period (remission), .

There were no deaths in the study group. There was no postoperative bleeding requiring reoperation, no anastomotic leakage/mechanical suture line (the most common complication reported in published studies thus far), one patient had postoperative wound infection (early complication), and two patients (7 and 9 months after surgery) required hospitalization due to abdominal pain, which resolved after conservative treatment. A patient had a BMI of 20 kg/m2 9 months after surgery. Due to weight loss of 30 kg (from 92 kg to 62 kg), the diagnosis was extended - no malabsorption was found, the diet was modified during the dietary consultation (the patient was not under the care of a dietician), and at the visit after 12 months the patient’s weight was 65.5 kg. One patient, due to intraoperative bleeding, was treated with conversion to the “open” method to manage bleeding; additionally, in the perioperative period, blood substitutes were transfused without complications. During follow-up visits to the Clinic, the patient’s haemoglobin and red blood cell count gradually normalized. In the 6th month after surgery, a postoperative hernia was diagnosed, which was treated laparoscopically with simultaneous cholecystectomy without complications.

### Basic descriptive statistics of the measured quantitative variables with the test of the normality of the distribution

Before starting to test the hypotheses, we computed basic descriptive statistics with the Shapiro-Wilk test. The distribution of most of the analyzed variables did not differ significantly from the Gaussian curve; however, the distributions of the variables % excess weight loss (EWL), qualifing HbA1c, fasting C-peptide and after after stimulation with glucagon differed statistically significantly from the normal distribution. Due to the small research sample, in further analyses, we mostly used nonparametric tests, such as the Mann-Whitney *U* test (Table 3).

**Table 3:** Basic descriptive statistics with a Shapiro-Wilk test

Variables	*M*	*Me*	*SD*	*Min*	*Max*	*p*
%EWL	105,21	101,18	40,14	56,94	241,73	0,001
%TWL	24,83	24,89	5,41	16,11	38,24	0,426
Age	50,18	53,50	10,42	29,00	66,00	0,175
Qualifying HbA1c	8,46	7,20	3,46	5,50	22,60	<0,001
Control HbA1c	6,11	6,01	0,98	4,69	8,94	0,142
Qualifying cholesterol	179,77	177,50	44,05	106,00	262,00	0,756
Control cholesterol	167,17	161,50	36,19	119,00	233,00	0,119
Qualifying triglycerydes	189,36	184,50	60,02	90,00	313,00	0,652
Control triglycerydes	127,17	106,50	72,54	67,00	377,00	<0,001
Qualifying LDL	104,59	105,50	39,91	45,00	185,00	0,526
Control LDL	92,00	86,00	30,93	45,00	149,00	0,060
Qualifying HDL	37,95	36,50	12,81	21,00	63,00	0,142
Control HDL	49,73	46,00	13,76	27,00	72,00	0,256
C-peptide fasting	1,95	1,82	0,87	1,00	4,08	0,027
C-peptide post-stimulation	3,09	2,62	1,66	1,29	7,64	0,015

*M*, mean; *Me*, median; *SD*, standard deviation; *Min*., minimal value; *Max*., maximal value

### Influence of selected factors on the recovery from type 2 diabetes

The results of a series of Mann-Whitney *U* tests indicated one difference at the level of statistical trend for the variable “age.” Patients who did not exhibit improvement were older (Median, Me = 56.00) than patients whose type 2 diabetes resolved (Me = 45.50). Next, the change in fasting and poststimulation C-peptide values were assessed by the division of patients into two analogous groups as in the analysis above. The results of the Wilcoxon tests showed statistically significant differences in both groups between fasting C-peptide and poststimulation C-peptide measurements. In both groups, the median value for fasting C-peptide was lower (improved group: Me = 2.11; unimproved group: Me = 1.72) than for poststimulation C-peptide (improved group: Me = 3.01; unimproved group: Me = 2.42) (Table 4).

**Table 4:** Analysis of differences between groups for selected variables using the *U* Mann-Whitney test

Variables	Type 2 diabetes no remission (*n* = 11)	Type 2 diabetes remission (*n* = 14)	*p*
	*Me*	*Me*	*p*
Age	56,00	45,50	0,062
Qualifying HbA1c	8,20	7,10	0,106
Qualifying cholesterol	167,00	178,00	0,639
Control cholesterol	152,50	164,00	0,747
Qualifying triglycerydes	206,00	163,00	0,953
Control triglycerydes	127,50	100,50	0,769
%EWL	101,31	100,14	0,661
%TWL	21,74	24,32	0,935
C-peptide fasting	1,72	2,11	0,406
C-peptide poststimulation	2,42	3,01	0,833

### Correlation of selected factors with HbA1c

The results showed one statistically significant correlation between LDL qualification and HbA1c qualification (correlation analyses using Spearman’s rho coefficient). This correlation was positive and moderately strong (0.3 < rho < 0.5), indicating that LDL values increase with increasing values for the HbA1c parameter (Table 5). We were also able to observe two correlations at the level of statistical trend for “qualifying HbA1c” and “qualifying cholesterol” and HDL. Both correlations were moderately strong. The negative relationship between HDL and HbA1c indicates that increasing HbA1C values are associated with decreasing HDL values.

**Table 5:** Analysis of the correlations between qualifying HbA1c and selected variables

Variables	Qualifying HbA1c
%EWL	0,26
%TWL	0,09
C-peptide fasting	−0,21
C-peptide poststimulation	−0,19
Age	0,08
Qualifying HDL	-0,40^
Qualifying LDL	0,46*
Qualifying triglycerydes	0,11
Qualifying cholesterol	0,35^

^^ 0,05 < *p* < 0,10; * *p* < 0,05; ** *p* < 0,1; *** *p* < 0,001^

### Correlation between fasting C-peptide and recovery from type 2 diabetes

Subjects whose c-peptide value was lower than the median cut-off value (Me = 1.75) were more likely to have no improvement in their condition (66.7% of all subjects with a low c-peptide result), whereas subjects who were included in the group with a high result of the index in question were more likely to experience improvement in their condition (72.7% of all subjects in the group in question).

## Discussion

The term “metabolic surgery” was first introduced in 1978 when Henry Buchwald and Richard Varco described it in their book, Metabolic Surgery [14]. This term defined surgery performed on normal organs or systems to achieve a biological effect leading to health improvement. Although work on the surgical treatment of type 2 diabetes has been performed previously [15], it was not until the late 1970s that surgery began to establish itself as an effective method of reducing weight, improving quality of life and facilitating the remission of many hitherto “incurable” diseases (including type 2 diabetes). In the present study, we evaluated the effectiveness and safety of surgical treatment of type 2 diabetes in patients with a BMI of 30–35 kg/m^2^. For this purpose, we qualified patients for OAGB surgery. The first OAGB was performed by Robert Rutledge in 1997 and the results were published in 2001 [16]. Since then, many papers (including randomised studies) have been published reporting the postoperative results of several thousand patients undergoing OAGB [17] and demonstrating the high efficacy and safety of this operation in its different variations [18, 19]. In terms of type 2 diabetes remission results, OAGB is a comparable surgery to Roux-en-Y gastric bypass (RYGB), and superior to restrictive bariatric surgery [20, 21]. The lower incidence of complications after OAGB and the greater technical ease of performing the operation [16, 22] than RYGB are highlighted.

We found published studies assessing the recovery of type 2 diabetes after surgery in patients with a BMI <35 kg/m^2^, but there are only a few reports in the world literature on the efficacy of Mini Gastric Bypass for the treatment of type 2 diabetes among patients with obesity of the first degree according to the WHO [23].

In one study, Lee and co-authors [24] evaluated the remission of type 2 diabetes after OAGB surgery among patients with a BMI <35 kg/m^2^. They found that fasting glucose levels returned to normal after 12 months among 89.5% of the operated patients, and the presumed end points of type 2 diabetes treatment (HbA1C<7.0%, LDL<150 mg/dl and triglycerides<150 mg/dl) were achieved in 76.5% of the operated patients.

In 2015, another paper was published on the surgical treatment of patients with type 2 diabetes and a BMI of 30–35 kg/m^2^. Kular et al. presented the results of 128 patients operated on at three Indian centers between 2007 and 2014 [25]. They assessed the the remission of type 2 diabetes (HbA1c <6% without pharmacotherapy) after 1, 2, and 7 years. They found complete remission among 64, 66, and 53% of the patients, respectively. The criteria for the diagnosis of obesity in patients of Asian origin are different from those in Europe (obesity of the first degree corresponds to a BMI of 27.5–32.4 kg/m^2^), and the results of the work of both Kular and Lee are not directly applicable to the European population.

The latest guidelines of the Society of American Gastrointestinal Endoscopic Surgery (SAGES) report the efficacy of surgical treatment of type 2 diabetes in a group with obesity of the first degree according to WHO [11].

In the vast majority of papers, the length of the excluded small bowel segment is 180-220 and is calculated according to the protocol proposed by Caballero et al. [26] (Table 6).

**Table 6:** The principle of calculating the length of the excluded section of the intestine in the OAGB (counted from the Treitz ligament)

Age	<35 years	35–50 years	>50 years
BMI <35 kg/m^2^	180 cm	160 cm	140 cm
BMI 35-40 kg/m^2^	200 cm	180 cm	160 cm
BMI 40-50 kg/m^2^	220 cm	200 cm	180 cm
BMI 55-60 kg/m^2^	240 cm	220 cm	200 cm
BMI >60kg/m^2^	1/2 intestine	1/2 intestine	1/2 intestine

Lee et al. performed surgery on 286 patients [12]. The patients were divided into three groups: 86 to the lower BMI group (BMI <40, mean 36.0), 286 patients in the middle BMI group (BMI 40-50, mean 43.2) and 72 patients in the higher BMI group (BMI> 50, mean 55.4). All procedures were performed laparoscopically. The average operative time was 130 min or the average hospital stay was 5.0 days. There were 23 minor early complications (4.3%) and 13 major complications (2.0%), including one fatal complication (0.016%). There were no significant statistical differences in operative time or complication rates between groups. The exclusion length was 150 cm for the first group, 250 cm for the second group, and 350 cm for the third group. The mean BMI reduction 2 years after surgery was 10.7, 15.5, and 23.3 kg/m^2^ for each group, respectively. Patients with a BMI <40 kg/m^2^ had more severe anaemia than patients in the other two groups. Patients operated on at the authors’ center did not have a diagnosis of type 2 diabetes (HbA1C 5.9–6.2%). In our work, we proposed the exclusion of 150 cm because of the lower risk of malnutrition; there will be papers in the literature about on the exclusion of such a segment of intestine, but not in the treatment of type 2 diabetes in patients in the European population, which is part of the innovation of this work. Mahawar et al. reported the results of 47364 operations performed by 118 surgeons worldwide [27]. Overall, 0.37% (138/36,952) of patients required revision surgery due to malnutrition. The highest rate of 0.51% (120/23,277) was recorded for some patients at an excluded small bowel length of > 200 cm, and the lowest rate of 0% was observed at a length of 150 cm.

To assess the effectiveness of this treatment, the changes in HbA1c value and dosage of medication are were used. In our study, the average HbA1c level before surgery was 8.45±3.3% and 12 months after surgery it was 6.16±0.96%, corresponding to a decrease of 2.29±3.3%. The remission rate of type 2 diabetes among patients with a BMI of 30-35 kg/m^2^ after metabolic exclusion and restriction surgery ranges from 50% to 100%. It depends on many factors, the most important of which are the type of surgery performed and the criteria adopted for disease remission. In his work, Busetto summarized the various definitions of remission [28]. When defined as a fasting blood glucose (<100 mg/dl), normal HbA1c (<6%) and no need for type 2 diabetes medication, the frequency was 66.3%. In contrast, when HbA1c was <7%, up to 80.0% of patients met these criteria. Reis et al. reported the highest proportion of remission of type 2 diabetes after OAGB (72.22%) and Roux-en-Y gastric bypass (70.43%) again providing support for the incretin theory and evidence for the effect of the exclusion of the proximal small bowel on the improvement of glycemia after surgery [29]. According to the accepted criteria, we found remission of type 2 diabetes among 14 patients (56%) and improvement among 6 (24%). Improvement among these patients meant complete discontinuation (*n*=4, from 40 to 80 units of insulin) or reduction of insulin doses (*n*=1, from 70 to 5 units per day) and continuation of oral hypoglycemic medication. Due to the place of residence of the patients (patients were from all over Poland), the modification of type 2 diabetes pharmacotherapy was the responsibility of family doctors/diabetologists in the location of residence. Despite maintenance of glycated haemoglobin <6.2% (6.04–6.19%) among another three patients in this group, the patients were maintained on oral hypoglycaemic medication (Glucophage XR). When asked by the attending physicians why the medication was not discontinued, they answered “just in case.” This is due to a certain “fear” of complete withdrawal of medication in diabetic patients, even though laboratory tests indicated complete remission of the disease. Cummings et al. also noted this phenomenon [30]. As highlighted in his paper, many physicians often leave patients on metformin therapy, even after normoglycaemia is achieved, to prevent relapse, with the hope of providing glycaemia-independent cardiovascular benefits or treatment of polycystic ovary syndrome. This practice contradicts any definition of type 2 diabetes remission, which requires patients to be off all antidiabetic medication. Unfortunately, there is no developed standard in bariatric/metabolic research on how to address this problem.

In the study group, we found no improvement among 5 patients, according to the accepted criteria. Despite the lack of improvement, patients discontinued (even those requiring >100 units of insulin per day) or significantly reduced their daily doses. Despite not meeting the criteria for “improvement,” patients benefited significantly from the associated weight reduction (%EWL > 80), reduced insulin doses and associated complications: hypoglycaemia, local reactions, postinsulin lipohypertrophy [31]. Patients in this group, despite the declaration of willingness to cooperate in the postoperative period expressed during the qualifying visits, did not follow the dietary recommendations and did not have regular follow-up visits.

The average body weight of patients undergoing surgery in our center was 97.1±11.6 kg, and 12 months after surgery, it was 73.8±12.2 kg, indicating a reduction in average BMI from 33.5±2 to 25.5±2.5 kg/m^2^, respectively, %EWL 102.7±38.5. These results are similar to those published in the literature to date and are unequivocally defined as a success of bariatric/metabolic surgery [32].

From October 2001 to October 2004, Wang et al. performed OAGB on in 423 consecutive patients (87 men and 336 women) due to morbid obesity [33]. The average age of the patients was 30.8 years, the preoperative average body weight was 120.3 kg, and the average BMI was 44.2 kg/m^2^. All operations were completed laparoscopically. The BMI decreased from 44.2 to 29.2 kg/m^2^ at 12 months after surgery, and the % EWL was 69.3%.

In their meta-analysis of the metabolic effects of bariatric surgery in type 2 diabetic patients with body mass index <35 kg/m^2^ Li et al. described a decrease in triglycerides after bariatric surgery of 56.67 mg/dl, cholesterol of 48.38 mg/dl, and LDL of 36.7 mg/dl, and an increase in HDL of 5.37 mg/dl [34]. In our study, despite the modification of the length of the excluded intestinal loop, the decrease in triglyceride values was much greater, and improvement in other lipid parameters was similar.

Statistical analysis of our patients’ results showed that the change in HbA1c resulting from surgery is correlated with preoperative LDL values, these results are not confirmed by other authors. Milone et al. evaluated changes in the lipid profile of patients after sleeve gastrectomy and OAGB [35]. At 12 months after surgery, the lipid profile as well as BMI and glycaemic control were better compared with baseline. Interestingly, these two surgical procedures were associated with completely comparable results at this time point, among patients after OAGB; specifically, % BMI showed a direct correlation with % TC and Δ% TG but not with Δ % HDLc and % LDLc.

The main argument against the use of OAGB has been the potential for bile reflux and its consequences [36]. However symptomatic bile reflux after OAGB is rare and cancer arising in the gastric pouch or oesophagus has never been reported after OAGB [37]. We have not found symptomatic bile reflux in patients operated on in our centre. As a standard, we did not perform gastroscopy as part of the follow-up one year after the operation.

Complications are an important part aspect of any surgical intervention. Among the patients undergoing OAGB in our center, one conversion to open surgery occurred (0.04%). In one patient, we found a wound infection in the postoperative period (0.04%). We did not find: anastomotic ulceration, anastomotic stenosis, or staple line bleeding, which are complications described in previously published studies. During the 12-month follow-up, no serious complications (deaths, severe nutritional deficiencies) were observed, and the identified postoperative complications could be effectively treated on an outpatient basis, leading to the conclusion that the level of complications in the observed group of patients is acceptable.

In their analysis, Yingjun et al. described the number of conversions (0.17–1.23%) and the most common complications after OAGB [38]. They divided complications into two groups: early perioperative complications such as bleeding (0.2–2.5%), staple line leak (0.1–2.1%) and wound infection (0.12–1.1%) and late complications, including reflux (0.9–2.0%), anastomotic ulceration (0.6–8%), and iron deficiency anaemia (4.9–8.1%), which can be treated conservatively. The perioperative mortality reported in this meta-analysis ranged from 0.08 to 0.18%. Comparing these results, the incidence of complications among patients undergoing surgery at our center was much lower compared with the incidence of complications in patients undergoing OAGB and the much more frequently performed Roux-en-Y gastric bypass [38, 39].

This study has some limitations. The main limitation is the relatively small sample of patients. At the start of the study, bariatric/metabolic operations in patients with first-degree obesity were financed by the national health fund in Poland, which changed during the study and reduced the eligibility of new patients.

## Conclusions

OAGB excluding 150 cm of the small intestine performed among patients with type 2 diabetes and obesity of the first degree (BMI 30–35 kg/m2), results in a complete withdrawal of insulin or a significant reduction in insulin doses and oral hypoglycaemic medication doses and, in more than half of patients, in complete recovery from type 2 diabetes. It also results in a significant reduction in body weight and improvement in blood pressure and lipid parameters. Younger age, fasting C-peptide, and C-peptide poststimulation levels yield better results.

An important element of “surgical treatment of type 2 diabetes” is, in addition to a well performed operation and appropriate qualification, systematic postoperative control (surgical, diabetic), compliance with dietary recommendations and patient involvement.

Due to the lower complication rate while maintaining high efficacy, OAGB with 150 cm of the small intestine excluded may be preferred in type 2 diabetes patients with obesity of the first degree.
